# A minimal model of *C. elegans* forward locomotion: the larval L1 circuit

**DOI:** 10.1186/1471-2202-12-S1-P42

**Published:** 2011-07-18

**Authors:** Jordan H Boyle, Netta Cohen

**Affiliations:** 1 School of Computing, University of Leeds, Leeds, West Yorkshire, LS2 9JT, UK; 2 Institute of Systems and Membrane Biology, University of Leeds, Leeds, West Yorkshire, LS2 9JT, UK

## 

The nematode Caernohabditis elegans possesses one of the simplest central nervous systems, and by far the best characterized. In the adult, the nervous system consists of a mere 302 neurons, of which the majority are located in the head, and the rest are distributed in the pharynx, along the ventral cord or in small ganglia. In particular, the local control (along the body) of the worm's sinuous undulations is instantiated by 8 classes of ventral cord motor neurons. But of those, only 3 classes exist as the larva hatches out of the egg (the so called L1 stage in development). This appears quite remarkable, given the similar repertoire of behaviors in the larva, as compared to the fully developed adult.

Here, we ask what minimal design principles must apply for the L1 circuit to generate robust forward locomotion. Robustness is taken to mean effective locomotion in different physical environments (e.g. high viscosity agar gels versus water) [1,2]. To address this challenge, we adopt an integrated neuromechanical model of forward locomotion in the adult that consists of the ventral cord nervous system, muscles, a body and a physical environment [2], and adapt the model nervous system and muscles to match the L1. In particular, we assume the forward locomotion control in the L1 consists of repeating units, each consisting only of a pair of neurons: one excitatory DB and one inhibitory DD. DB neurons are modeled as bistable elements [2] innervating dorsal muscles, and DD neurons are modeled as linear elements inhibiting ventral muscles. High level control is modeled with an on/off AVB unit that drive all B-neurons. The rhythm is generated entirely on the dorsal side and is achieved by a closed-loop proprioceptive feedback (via postulated stretch sensitivity of DB neurons). Inhibitory neurons receive synaptic inputs from the local DB neuron and are therefore enslaved to its oscillations. The integrated model successfully generates and propagates coordinated undulations on agar and liquid model environments. In the model, both of these behaviors are achieved by a single (i.e., fixed-parameter) model worm.

The model makes a number of relatively easily testable predictions:

1) L1 larva have a minimal, reflex-driven mechanism to control body undulations.

2) Inhibitory D-neurons are essential for forward locomotion in the L1.

3) Muscle properties must differ substantially between the ventral and dorsal side (specifically, ventral but not dorsal muscles must be spontaneously active).

## References

[B1] Berri S Boyle JH Tassieri M Hope IA Cohen N Forward locomotion of the nematode *C. elegans* is achieved through modulation of a single gait HFSP Journal 2009 3 3 186 193 19639043 10.2976/1.3082260 PMC2714959

[B2] Boyle JH PhD thesis 2009 University of Leeds, Leeds

